# Etherification of Glycerol with Propylene or 1-Butene for Fuel Additives

**DOI:** 10.1155/2017/4089036

**Published:** 2017-08-03

**Authors:** Chakrapong Saengarun, Amorn Petsom, Duangamol Nuntasri Tungasmita

**Affiliations:** ^1^Program of Petrochemistry, Faculty of Science, Chulalongkorn University, Bangkok 10330, Thailand; ^2^Department of Chemistry, Faculty of Science, Chulalongkorn University, Bangkok 10330, Thailand; ^3^Materials Chemistry and Catalysis Research Unit, Department of Chemistry, Faculty of Science, Chulalongkorn University, Bangkok 10330, Thailand

## Abstract

The etherification of glycerol with propylene over acidic heterogeneous catalysts, Amberlyst-15, S100, and S200 resins, produced mono-propyl glycerol ethers (MPGEs), 1,3-di- and 1,2-di-propyl glycerol ethers (DPGEs), and tri-propyl glycerol ether (TPGE). The propylation of glycerol over Amberlyst-15 yielded only TPGE. The glycerol etherification with 1-butene over Amberlyst-15 and S200 resins produced 1-mono-, 2-mono-, 1,2-di-, and 1,3-di-butyl glycerol ethers (1-MBGE, 2-MBGE, 1,2-DBGE, and 1,3-DBGE). The use of Amberlyst-15 resulted in the propylation and butylation of glycerol with higher yields than those obtained from the S100 and S200 resins. The PGEs, TPGE, and BGEs were evaluated as cold flow improvers and octane boosters. These alkyl glycerol ethers can reduce the cloud point of blended palm biodiesels with diesel. They can increase the research octane number and the motor octane number of gasoline.

## 1. Introduction

Biodiesel is manufactured from vegetable oils or animal fats by transesterification between the fatty acids and methanol, which converts them into fatty acid methyl esters with glycerol as a coproduct. The mass production of biodiesel would entail surplus glycerol production. One possible way to utilize glycerol is as glycerol-based fuel additives. The transformation of glycerol into fuel oxygenates has been developed using the etherification of glycerol with alkenes or alcohols, the esterification of glycerol with acetic acid, and the ketalization of glycerol and ketones, from which polyglycerols, alkyl glycerol ethers, acetyl glycerols, and solketal were obtained, respectively [1, 2]. The conversion of glycerol into glycerol-based additives has been developed by using different types of catalysts. The selective etherification of glycerol over alkaline earth metal oxides gave polyglycerols [3]. The alkyl glycerol ethers from the etherification of glycerol with alkenes or alcohols were catalyzed by acid ion exchange resins [4–6], sulfonic acid functionalized mesostructured silicas [7], perfluoropolymer supported silicas [8], rare-earth-modified zeolites [9], and acid-treated zeolites [10]. Heterogeneous solid catalysts such as sulfonic acid functionalized mesostructured silicas [11] and acid ion exchange resins and zeolites [12] have been studied in the esterification of glycerol with acetic acid. The ketalization of glycerol with acetone to solketal catalyzed by zeolites [13], acid ion exchange resins [14], and sulfonic acid [15] has also been studied.

These glycerol-based fuel additives were tested as cold flow improvers on the viscosity of biodiesel [15–19]. It was reported previously that the glycerol-based additive solketal improved the octane number and reduced gum formation in gasolines [20]. The blending of glycerol-based additives with diesel fuel has reduced the amount of particulate emissions, increased the lubricity of diesel, increased engine performance, and increased the distillation temperature of diesel [21–24]. Moreover, these additives also show increased performance for the wear preventive characteristics of lubricating grease [13].

The aim of this work is to study the etherification of glycerol with propylene or 1-butene over commercial heterogeneous acid catalysts, that is, Amberlyst-15 and S100 and S200 ion exchange resins. In addition, this work aims to investigate the obtained alkyl glycerol ethers for their potential use as cold flow improvers of palm biodiesel blended with diesel, as cetane number improvers of diesel and palm biodiesel, and as octane number improvers of gasoline.

## 2. Materials and Methods

### 2.1. Materials

The chemicals used in this study were glycerol 99.78% and biodiesel 97.62%, which were supplied by PTT Global Chemical Public Company Limited, Thailand. Propylene 99.58%, 1-butene 99.84%, diesel with 34 ppm sulfur, and gasoline with 2.8% polycyclic aromatic hydrocarbons were supplied by IRPC Public Company Limited, Thailand. The sulfonic acid functionalized styrene divinyl benzene copolymers, S100 and S200, were supplied by Bayer. Wet S100 with brown gel-type beads of 0.58 mm with uniform particle size and wet S200 with dark brown gel-type beads of 0.60 mm for the mean bead size were washed with deionized water and then with 15% HCl and rinsed with deionized water. The effluent pH was measured until it became higher than 4.3, and then the resin was dried at 110°C for 12 h. Amberlyst-15, a strong acid ion exchange resin bead of 0.60–0.80 mm with a pale gray color, was supplied by Rohm and Hass. The acid capacity of the catalysts was measured by the following procedure: 0.05 g of the catalyst was added to 15 g of 2 M NaCl. The suspension was equilibrated and then was titrated with 0.01 M NaOH aqueous solution [25].

### 2.2. Etherification of Glycerol with Propylene or 1-Butene

The etherification reactions of glycerol with propylene or 1-butene were performed in a stainless steel home-built micro reactor that was equipped with a pressure gauge and a magnetic stirrer. The etherification reactions of glycerol with propylene or 1-butene were performed by the following procedure. Glycerol (1 mol) and 7 to 12 wt% of the catalyst based on glycerol were added to the reactor. Nitrogen gas was used to purge the reactor 3 times to eliminate oxygen. In each experiment, liquefied olefin gas, 2 to 5 mol of propylene or 1-butene, was introduced into the reactor. The initial reaction conditions were adjusted to the following: the starting pressure was increased to 20 bars by the addition of nitrogen gas, the temperature was 100°C, and the stirring speed was set at 1,000 rpm. The reaction times varied (8, 16, 24, 40, 48, and 72 h). The reactor was cooled at room temperature after completion of the reaction.

The etherification products were analyzed using gas chromatography (GC), Agilent model 7890A, which was equipped with a DB-624 column (60 m, 0.25 mm, and 1.4 *μ*m), and a mass spectrometric detector (MS), Agilent model 5975C. The carrier gas was helium. Analysis was carried out with a temperature program from an initial temperature of 45°C with a holding time of 5 min to a final temperature of 220°C with a holding time of 10 min and a temperature rate change of 20°C/min. The temperatures of the injector and detector were controlled at 240°C. The etherification products were characterized for the chemical structure and quantitative chemical composition using mass spectra and integrated peak area normalization, respectively. The products from the etherification reaction were prepared before introduction into the GC by diluting 50 mg/ml of the product in methanol [26]. The conversion of glycerol was defined by

(1) $$\begin{array}{c} \text{Conversion }\left(\;\%\;\right)=\frac{\left[\text{converted glycerol}\times 100\right]}{\left[\text{total glycerol}\right]}. \end{array}$$

### 2.3. Effectiveness of Alkyl Glycerol Ethers as Cold Flow Improvers and Fuel Additives

PGEs and TPGE from the etherification of glycerol with propylene and BGEs from the etherification of glycerol with 1-butene were evaluated as fuel additives. These alkyl glycerol ethers were added to palm biodiesel and palm biodiesel blended with diesel B2 (2% biodiesel), B5, B80, B90, and B100 for the determination of the cloud point according to the procedures of the American Society for Testing and Materials (ASTM) D 2500 [27]. The experiments were performed at different concentrations of the additive: 0.1, 0.5, 1, 2, 3, 4, 5, 6, 7, 8, 9, and 10%. The additives were added to the diesel and palm biodiesel for the determination of the cetane number (CN) according to the procedure of ASTM D 613 [27]. The experiments were performed with different oxygenated additives, that is, ethanol, methyl tertiary butyl ether (MTBE), PGEs, TPGE, and BGEs, with 10% of each oxygenated additive added to diesel and palm biodiesel. The research octane number (RON) and motor octane number (MON) in gasoline blended with 10% of each of the oxygenated additives (ethanol, MTBE, PGEs, TPGE, and BGEs) were determined according to ASTM D 2699 and D 2700, respectively [27].

## 3. Results and Discussion

### 3.1. Characterization of Etherification Products Using GC-MS

**Figure 1:**
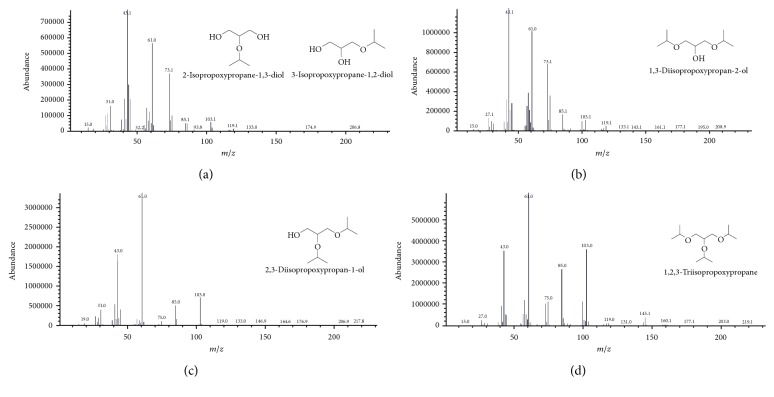
Mass spectra of propyl glycerol ethers: (a) 1-MPGE and 2-MPGE, (b) 1,3-DPGE, (c) 1,2-DPGE, and (d) TPGE.

As shown in Figure 1(a), 1-MPGE (or 3-isopropoxypropane-1,2-diol) and 2-MPGE (or 2-isopropoxypropane-1,3-diol) were identified by the same peak of the chromatogram. The ion of the hydroxyl methyl group ([CH_2_OH]^+^ at peak *m*/*z* = 31) was determined to indicate the presence of the primary alcohol group in the structure of the MPGEs. The propyl fragment ion ([C_3_H_7_]^+^ at peak *m*/*z* = 43) from the molecular ions was identified as the base peak. The ethyl diol group was eliminated from the molecular ion that gave a fragmented ion of methyl propyl ether ([M-C_2_H_5_O_2_]^+^ at peak *m*/*z* = 73). The fragmented ions of [C_3_H_7_O_2_]^+^ at peak *m*/*z* = 75, [C_5_H_9_O]^+^ at peak *m*/*z* = 85, and [C_5_H_11_O_2_]^+^ at peak *m*/*z* = 103 were also present in the spectrum of 1,3-DPGE (1,3-diisopropoxypropan-2-ol) in Figure 1(b), 1,2-DPGE (2,3-diisopropoxypropan-1-ol) in Figure 1(c), and TPGE (1,2,3-triisopropoxypropane) in Figure 1(d). The methyl propyl ether group was eliminated from the TPGE that gave [M-C_4_H_9_O]^+^ at peak *m*/*z* = 145.

**Figure 2:**
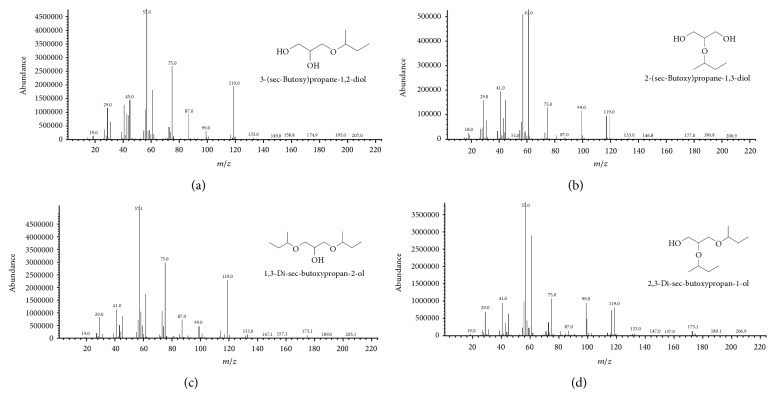
Mass spectra of butyl glycerol ethers: (a) 1-MBGE, (b) 2-MBGE, (c) 1,3-DBGE, and (d) 1,2-DBGE.

In previous work, the mass spectrum of 3-tert-butoxy-propane-1,2-diol showed a corresponding ion of a tert-butyl group, suggesting the presence of primary and tertiary alcohol groups [28]. As shown in Figure 2, there were four components of butyl glycerol ethers (BGEs) in the etherification products of glycerol with 1-butene over Amberlyst-15. The spectrum of 1-MBGE or 3-(sec-butoxy)propane-1,2-diol, Figure 2(a), showed the molecular ions of [C_3_H_7_O_2_]^+^, [C_5_H_11_O]^+^, and [C_5_H_11_O_3_]^+^ that were detected at peaks *m*/*z* = 75, 87, and 119, respectively. The 2-MBGE or 2-(sec-butoxy)propane-1,3-diol, Figure 2(b), exhibited the elimination of a hydroxyl methyl group [M-CH_3_O]^+^ at peak *m*/*z* = 117. The spectrum of 1,3-DBGE or 1,3-di-sec-butoxypropan-2-ol, Figure 2(c), showed the elimination of an ethyl group from the molecular ion, which gave [M-C_2_H_5_]^+^ at peak *m*/*z* = 175. The 1,2-DBGE or 2,3-di-sec-butoxypropan-1-ol, Figure 2(d), presented a peak at *m*/*z* = 173, which corresponded to the elimination of a hydroxyl methyl group [M-CH_3_O]^+^.

### 3.2. Reaction Results

#### 3.2.1. The Effect of Reaction Temperature

**Figure 3:**
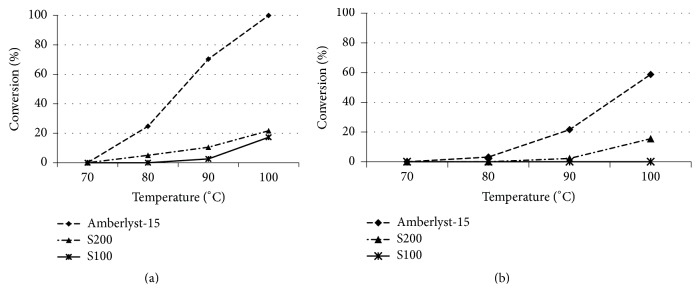
Influence of temperature on glycerol etherification with propylene (a) and 1-butene (b) [glycerol : olefin = 1 : 4, catalyst 10 wt% based on glycerol, 24 h]. The data are shown as the mean ± 1.88 SD derived from 3 independent samples.

The dependence of glycerol conversion on the reaction temperature was examined. The experiments using Amberlyst-15, S100, and S200 catalysts were conducted at 70, 80, 90, and 100°C. As shown in Figure 3(a), the highest conversion of glycerol propylation was obtained at 100°C after 24 h over Amberlyst-15. However, the results show that the conversion increased slightly from 80 to 100°C after 24 h over S100 and S200 resins. As shown in Figure 3(b), the conversion of glycerol butylation slightly increased with increasing temperature from 80 to 100°C after 24 h over Amberlyst-15 and S200.

#### 3.2.2. The Effect of Catalyst Loading

**Figure 4:**
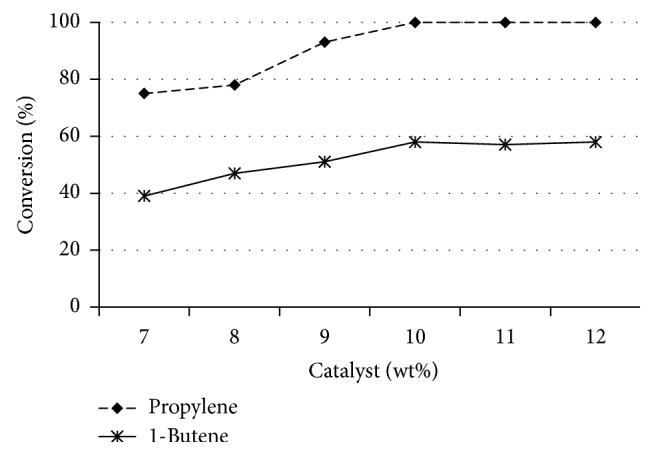
Influence of catalyst loading on glycerol etherification with olefin [glycerol : olefin = 1 : 4, 24 h, at 100°C]. The data are shown as the mean ± 2.98 SD derived from 3 independent samples.

When Amberlyst-15, S100, and S200 were used as the catalysts, the effect of catalyst loading on glycerol etherification with propylene or 1-butene was investigated with various loads in the range of 7 to 12 wt% based on glycerol. As shown in Figure 4, the conversion of glycerol propylation and butylation increased with the increase of the catalyst from 7 to 10 wt% and then the conversion was constant with the catalyst load from 10 to 12 wt%. A sufficient amount of catalyst can accelerate the etherification. Therefore, the optimal catalyst dosage was chosen to be 10 wt% based on glycerol.

#### 3.2.3. The Effects of the Molar Ratio of Glycerol/Olefin

**Figure 5:**
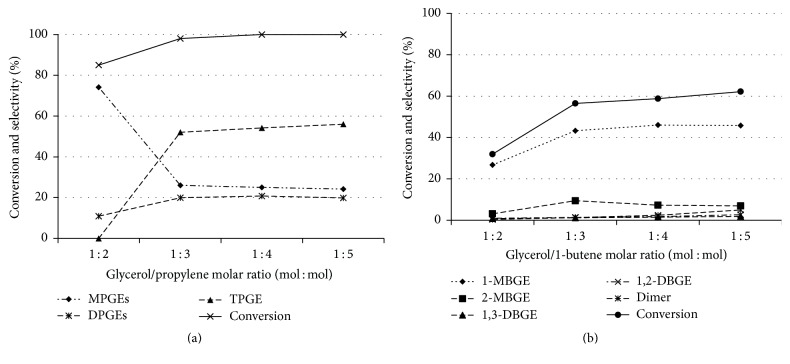
Effect of the molar ratio of glycerol/propylene (a) and glycerol/1-butene (b) on glycerol etherification [catalyst 10 wt% based on glycerol, 24 h, at 100°C]. The data are shown as the mean ± 1.64 SD derived from 3 independent samples.

The effect of the glycerol to propylene or 1-butene molar ratio on the etherification reaction was studied using four different ratios within the range of 1 : 2 to 1 : 5.  Figure 5(a) illustrates the impact of the glycerol to propylene molar ratio on the glycerol conversion and selectivity of PGEs. With a glycerol to propylene molar ratio of 1 : 2, the formation of MPGEs and DPGEs occurred. Within the increment of 1 : 4 to 1 : 5 for the glycerol to propylene molar ratio, the glycerol conversion was enhanced and the selectivity towards TPGE in the ether mixture increased. Considering the glycerol to propylene molar ratio of 1 : 4, appropriate conditions were the catalyst at 10 wt%, based on glycerol, for 24 h at 100°C. As shown in Figure 5(b), increasing the glycerol to 1-butene molar ratio from 1 : 3 to 1 : 5 caused the glycerol conversion and the selectivity towards MBGEs and DBGEs to slightly increase. The dimerization of 1-butene is an undesired side reaction that depends on the molar ratio. The glycerol to 1-butene molar ratio of 1 : 5 produced more di-1-butene (dimer). Therefore, a glycerol to 1-butene molar ratio of 1 : 4 with the catalyst at 10 wt% based on glycerol for 24 h at 100°C was chosen in subsequent experiments.

#### 3.2.4. The Effects of Reaction Time

The influence of the reaction time on the glycerol conversion and alkyl glycerol ether selectivity was investigated. Experiments using the same amount of catalyst, 10 wt%, were carried out at 8, 16, 24, 40, 48, and 72 h. The results are presented in Tables 1 and 2. As shown in Table 1, ion exchange resins S100 and S200 gave lower catalytic activity than Amberlyst-15. The propylation of glycerol over resins S100 and S200 could not reach complete conversion of glycerol after 72 h. The reaction rates over resins S100 and S200 was boosted up slowly from 16 to 48 h and reached 69.92% over S100 and 72.43% over S200 after 72 h. The selectivity of alkyl glycerol ethers was also affected by the reaction time. The product distribution of the propylation of glycerol over the S100 resin gave a similar result to what was obtained over the S200 resin. The MPGEs were the main products in this reaction, while the DPGEs were at the lowest composition when using S200 and S100 as the catalysts. The Amberlyst-15 gave the best performance in the propylene alkylation of glycerol in which glycerol reached 100% conversion and the reaction reached equilibrium after 24 h at 100°C. The equilibrium yields of MPGEs, DPGEs, and TPGE were 24.99%, 20.84%, and 54.17%, respectively. The transformation of TPGE at 100% was obtained by increasing the reaction time to 48 h. Considering both glycerol conversion and PGE selectivity over Amberlyst-15, 24 h of reaction time was appropriate. In previous study, the strong acid resins were very active catalysts for etherification between glycerol and isobutylene. The reaction achieved 100% glycerol conversion with 92.7% selectivity to di- and triethers with 4.9% of di-isobutylenes, as a side reaction, over Amberlyst-39 after 8 h reaction time at 60°C. Under the same reaction conditions, Amberlyst-15 gave 86.6% selectivity to di- and triethers with the 10.9% production of di-isobutylenes [29]. The etherification of glycerol with isobutylene in the previous study used a lower reaction time and temperature when compared with the etherification of glycerol with propylene over acid resins in this research. Interestingly, the propylation of glycerol gave a sole product of tri-propyl glycerol ether when Amberlyst-15 was utilized.

The results of glycerol conversion and the product distribution for 1-butene alkylation of glycerol over Amberlyst-15 and S200 are shown in Table 2. The catalyst S100 in the etherification of glycerol with 1-butene gave no activity. The 1-butene alkylation of glycerol over Amberlyst-15 reached equilibrium after 72 h at 100°C. The equilibrium yields of 1-MBGE, 2-MBGE, 1,3-DBGE, 1,2-DBGE, and dimer were 66.29%, 7.80%, 9.71%, 10.89%, and 5.31%, respectively. The identification of chemical structures from the interpretation of mass spectra confirmed that TBGE was not formed in the etherification reaction between glycerol and 1-butene. Under the same conditions, the S200 resin reached a glycerol conversion of only 38.99%. The dimerization of 1-butene depends on the reaction temperature and time. A higher reaction time would promote the dimerization reaction, which would consume a large amount of 1-butene and would influence the glycerol selectivity towards DBGEs. Considering both the glycerol conversion and BGE selectivity over Amberlyst-15, 72 h of reaction time was appropriate.

**Scheme 1:**
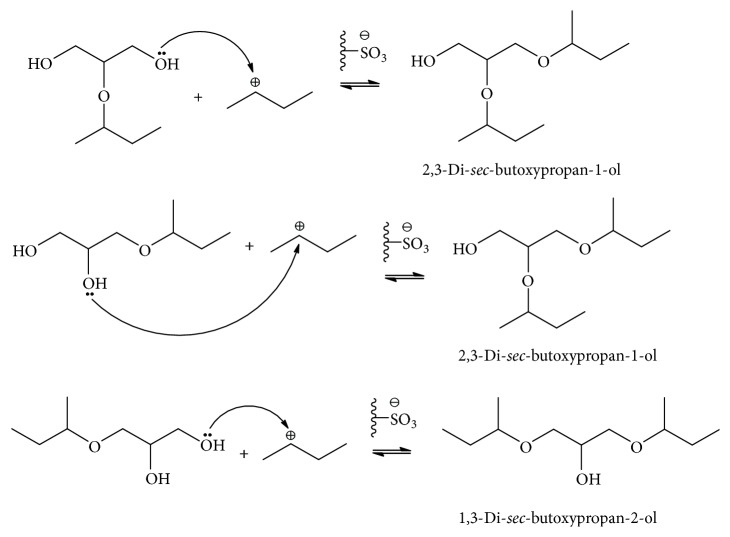
Reaction pathway of etherification of 1-MBGE and 2-MBGE with 1-butene on Amberlyst-15 to form 1,2-DBGE and 1,3-DBGE.

These results can be explained as follows. In etherification reactions, thermal and acidic conditions will protonate the double bonds of propylene or 1-butene to be electron acceptors (Lewis acid). Amberlyst-15 exhibited the highest catalytic performance because of its high acidity of 5.22 mmol/g compared with 3.53 mmol/g for the S200 resin and 3.00 mmol/g for the S100 resin. The –OH groups of the glycerol molecule are the electron donor groups (Lewis base). This means that –OH end group of the glycerol reacted rapidly with the isopropyl cation or 2-butyl cation to form the carbon-oxygen bond of the 1-monoether. Then another cation intermediate formed a new covalent bond with an unshared pair of electrons from the oxygen atom of a second –OH group, which could be either that in position 2 (1,2-diether) or that in position 3 (1,2- or 1,3-diether). Finally, for steric reasons or for higher activation energy of butyl cation, only isopropyl cation could form the remaining carbon-oxygen covalent bond for TPGE (Scheme 1).

#### 3.2.5. Reusability of the Catalyst

**Figure 6:**
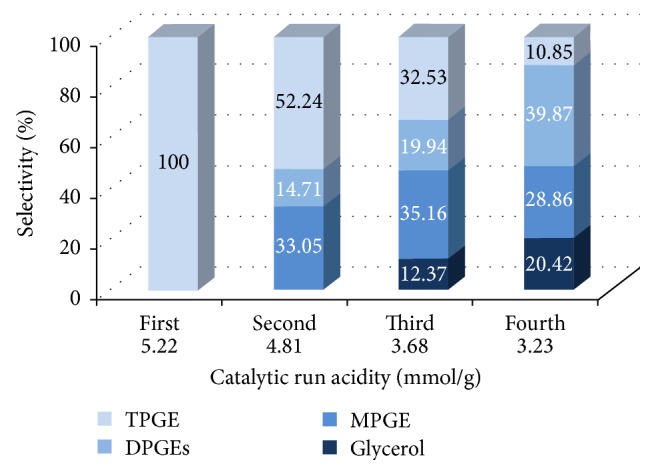
Catalyst reuse: product distribution of etherification of glycerol with propylene, in four sequential catalytic runs reusing Amberlyst-15 with acidity measurement [glycerol : propylene = 1 : 4, catalyst 10 wt% based on glycerol, temp 100°C, 48 h]. The data are shown as the mean ± 2.39 SD derived from 3 independent samples.

**Figure 7:**
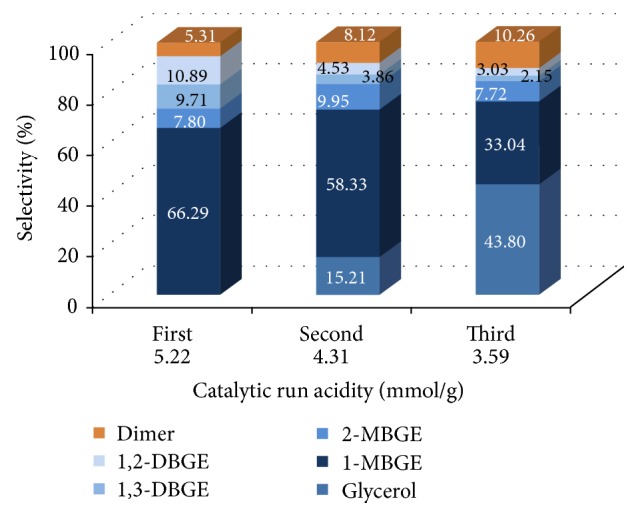
Catalyst reuse: product distribution of etherification of glycerol with 1-butene in three sequential catalytic runs reusing Amberlyst-15 with acidity measurement [glycerol : 1-butene = 1 : 4, catalyst 10 wt% based on glycerol, temp 100°C, 72 h]. The data are shown as the mean ± 1.81 SD derived from 3 independent samples.

After each catalytic run, the catalyst was recovered by filtration, washed with ethanol, and dried by air. Before use of the catalyst in each run, the acid capacity of the used catalyst was measured using the same procedure as for the fresh catalyst. Amberlyst-15 has been reused in the propylation and butylation of glycerol with the optimal reaction conditions in order to evaluate life time of the catalyst.  Figure 6 indicates the performance of reused Amberlyst-15 in four sequential catalytic runs of glycerol propylation after 48 h at 100°C. The results show that catalytic performance was lost in either the conversion of glycerol or the product distribution. The performance of reused Amberlyst-15 in three sequential catalytic runs of glycerol butylation, after 72 h at 100°C, is indicated in Figure 7. The lower acid capacity of the catalyst during the glycerol butylation after 72 h would increase the dimerization reaction of 1-butene that influences the glycerol selectivity towards MBGEs and DBGEs. The catalytic performance, glycerol conversion, product distribution, and side reaction (for glycerol butylation) depended on the acid capacity of reused Amberlyst-15.

**Figure 8:**
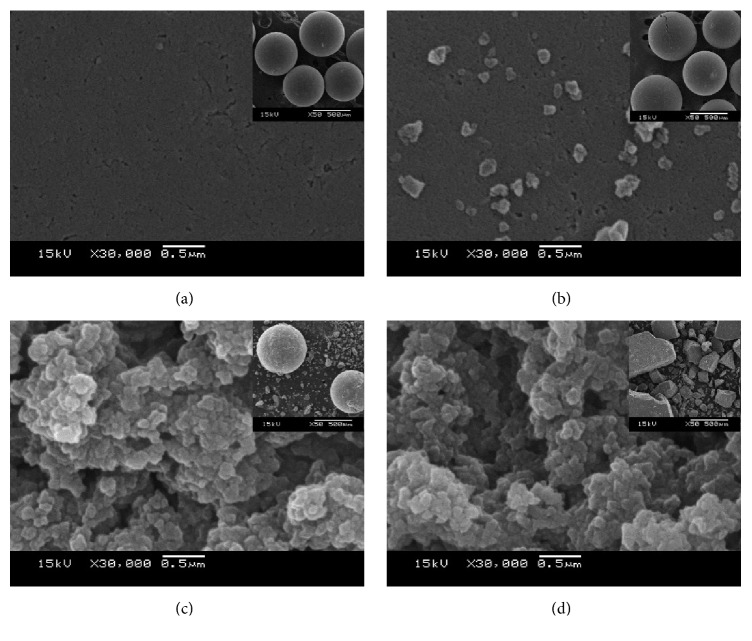
SEM of (a) fresh Amberlyst-15 (b) after first run, (c) after second run, and (d) after third run.

The catalyst deactivation of the Amberlyst-15 after the sequential catalytic runs was coke deposition and loss of acidic functional groups from its surface. The surface images of the catalysts were examined using scanning electron microscopy (SEM) at the magnification of 30,000x at the scale of 0.5 *μ*m and 50x at the scale of 500 *μ*m for inset figure as shown in Figure 8. The SEM image of the second sequential catalytic run showed coke deposition on a smooth surface as shown in Figure 8(b). The surfaces of the third and fourth used Amberlyst-15 showed a cracked surface with coke deposition as shown in Figures 8(c) and 8(d).

**Figure 9:**
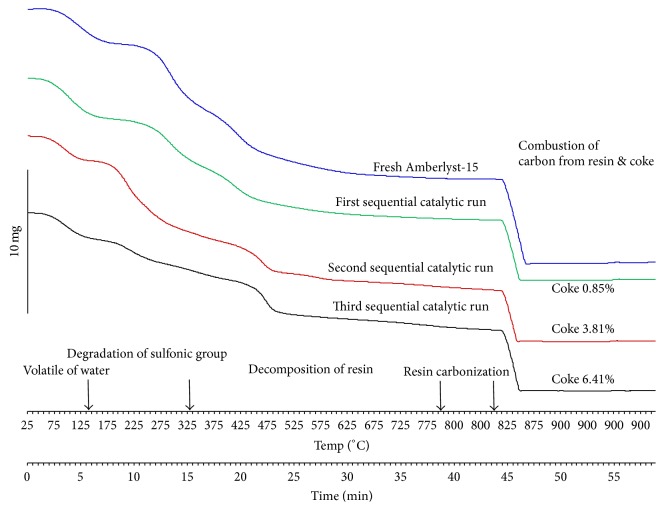
TGA of the Amberlyst-15 before and after the sequential catalytic runs.

Furthermore, the quantity of coke on the reused Amberlyst-15 was determined using thermogravimetric analysis (TGA). The TGA of the Amberlyst-15 before and after the sequential catalytic runs was carried out following the temperature profile of 25–800°C under nitrogen atmosphere at a heating rate of 20°C/min. An isothermal temperature at 800°C was held on for 5 min. Then, temperature was raised up from 800 to 900°C with a heating rate of 20°C/min. Finally, the isothermal temperature was controlled at 900°C for 10 min under oxygen atmosphere as shown in Figure 9. The TGA curve of the Amberlyst-15 indicates the loss of water from the catalyst at 25 to 140°C. The second weight loss between 140°C and 330°C was degradation of sulfonic functional group. The third weight loss from 330°C to 800°C was decomposition of styrene divinyl benzene resin. The remaining material was carbon from resin carbonization [30], which was still left at 800°C. The carbon from resin was combusted at 900°C. The amount of coke was deposited in the catalysts after the first, the second, and the third catalytic runs as 0.85%, 3.81%, and 6.41%, respectively.

#### 3.2.6. Reaction Performance

In previous study, the transformations of glycerol into butyl glycerol ethers have been developed by using etherification of isobutylene [1, 2, 7–10, 29]. The product selectivity gives the mixture of mono-, di-, triethers and dimer. This work intends to study the etherification of glycerol with propylene or 1-butene in which these reactants are available from petrochemical industry. These reactions have never been report. The product selectivity of etherification between glycerol and propylene achieved 100% of triether without dimerization. At the conditions in this study, the conversion of glycerol over Amberlyst-15 was 100%. In comparison with other studied catalysts, Amberlyst-15 was active enough and only 5.31% of 1-butene dimer was created in reaction mixture [1].

### 3.3. Influence of PGEs, TPGE, and BGEs on the Cold Flow Property of Palm Biodiesel

Generally, the palm biodiesel contains 45.6% saturated fatty acids (palmitic acid and stearic acid), 42.7% monounsaturated fatty acids (oleic acid), and 10.3% polyunsaturated fatty acids (linoleic acid) [31]. They form the cluster of hydrocarbon crystals in the blended fuels and begin to build up a gel layer that is visually observed when the blended fuels are cooled. In the previous work, the ethyl glycerol ethers were obtained at 180°C from the glycerol reaction with ethanol with a molar ratio of 1 : 3 after 4 h over the Amberlyst-15 catalyst. Laboratory tests using a blend containing 0.5–1.0% of these ethyl glycerol ethers in biodiesel showed a reduction of the cloud points. The reduction in the cloud point is 2 and 4°C for soybean and tallow B100, respectively [6]. To obtain the efficiency of PGEs, TPGE, and BGEs as cold flow improvers, the performance of their blends with palm biodiesel and palm biodiesel blended with diesel was evaluated. PGE was collected from the propylation of glycerol over Amberlyst-15 for 24 h, which reached 100% glycerol conversion. TPGE was collected from the propylation of glycerol over Amberlyst-15 for 48 h. BGE was selected from the butylation of glycerol over Amberlyst-15 for 72 h, which reached 100% glycerol conversion. These three oxygenated compounds had the best solubility in blended palm biodiesels. The cold flow temperatures of blended palm biodiesels decreased according to the increase in the concentrations of these additives. The cloud points of blended palm biodiesels (B2, B5, B80, B90, and B100) were reduced in the ranges of 1–3°C, 3-4°C, and 4–7°C when PGEs were added to the blended palm biodiesels at concentrations in the range of 0.1–1%, 2–6%, and 7–10%, respectively. The blended concentrations of TPGE at 0.1–1%, 2–6%, and 7–10% decreased the cloud point of the blended palm biodiesels in the ranges of 1-2°C, 2-3°C, and 3–7°C, respectively. Similarly, the cold flow temperatures of the blended palm biodiesels were decreased in the ranges of 1–5°C, 5–7°C, and 7–13°C by adding BGEs at blended concentrations of 0.1–1%, 2–6%, and 7–10%, respectively. These results demonstrate that PGEs, TPGE, and BGEs achieved an improvement in the cold flow property of palm biodiesels and their blends with diesel. There are hydrocarbon chains in palm biodiesel, that is, palmitate and stearate, which have hydrophobic regions. However, the methyl ester group in palm biodiesel has a hydrophilic region. It should be noted that when the palm biodiesel was cooled, the polar groups of PGEs, TPGE, and BGEs would come in contact with the polar methyl ester groups to reduce and arrange the cluster size of the hydrocarbon crystals. Thus, the cold flow temperatures were reduced when the crystal size of the saturated fatty acids was decreased [32].

### 3.4. The Influence of PGEs, TPGE, and BGEs on the Cetane and Octane Numbers of Fuels

ASTM D 6751 defines a cetane number for biodiesel from 48 to 65 for satisfactory diesel engine operation. Higher cetane numbers help ensure good cold start properties and minimize the formation of white smoke. The ASTM limit for the B100 cetane number is set to 47 because this is the level identified for premium diesel fuel [32]. Previous tert-butyl glycerol ether research work reported that 10% of ethers (MBGEs 1.6%, DBGEs 39.2%, and TBGE 58.3%) blended in diesel could decrease the cetane number by 2.5 points [8]. The cetane number of diesel and palm biodiesel in this paper was measured after adding 10% of each oxygenated-compound additive, that is, ethanol, MTBE, PGEs, TPGE, and BGEs, which are shown in Table 3. PGEs, TPGE, and BGEs can reduce the cetane number of diesel and palm biodiesel. However, MTBE increased the cetane number of diesel and palm biodiesel. The results indicate that ethanol, PGEs, TPGE, and BGEs reduce 0.1, 3.0, 5.7, and 2.2 points from the cetane number of diesel, respectively. However, the cetane number of diesel was increased 1.4 points by MTBE. It was also reported that 1.3, 0.4, and 0.8 points from the cetane number were reduced by blending PGEs, TPGE, and BGEs in palm biodiesel, respectively. However, ethanol and MTBE increased the cetane number of palm biodiesel by 1.5 and 2.7 points, respectively. It could be noted that PGEs, TPGE, and BGEs could adjust the high cetane number of diesel and palm biodiesel for the control value.

Oxygenated compounds help gasoline to burn more completely. Other glycerol etherification research work reported that the obtained glycerol ether has octane numbers of 112–128 for the RON and 91–99 for the MON which is suitable for a gasoline component [33]. In previous work, the solketal from the reaction of glycerol with acetone increased the octane number by up to 2.5 points for gasoline [20].  Table 4 indicates the performance of the oxygenated-compound additives, that is, ethanol, MTBE, PGEs, TPGE, and BGEs, in the octane number of gasoline. PGEs and TPGE gave an octane number lower than MTBE and ethanol. The results indicate that PGEs and TPGE can increase the RON in gasoline by 0.5 and 1.0, respectively. The MON in gasoline increased by 3.0 and 2.6 when PGEs and TPGE were blended into gasoline, respectively. Furthermore, BGEs gave a higher MON than MTBE. It should be concluded that the new oxygen-containing compounds, PGEs and TPGE from glycerol etherification with propylene and BGEs from glycerol etherification with 1-butene, could be used as fuel supplements because they increased the numerical rating of the knock resistance and increased the oxygen content in gasoline for complete combustion.

## 4. Conclusion

The etherification of glycerol with propylene or 1-butene over acidic heterogeneous catalysts, Amberlyst-15, S100, and S200, at 100°C, was carried out. The results indicate that Amberlyst-15 exhibits the highest activity on propylene or 1-butene. The product of the propylation of glycerol was TPGE, which reached a complete formation through the propylene alkylation of MPGEs and DPGEs. The products from the glycerol etherification with 1-butene were 1-MBGE, 2-MBGE, 1,2-DBGE, and 1,3-DBGE. These reactions showed that propylene could perform with a shorter reaction time compared to the reaction of 1-butene.

The PGEs and BGEs reduced the cold flow temperature of blended palm biodiesels. Moreover, they increased the RON and MON in gasoline. The increase in the numerical rating of the knock resistance of these alkyl glycerol ethers confirmed that these materials could be used as an alternative fuel supplement.

## Figures and Tables

**Table 1:** Product distribution for the etherification of glycerol with propylene on Amberlyst-15, S-200, and S-100 catalysts [glycerol : propylene = 1 : 4, catalyst 10 wt% based on glycerol, at 100°C].

Time (h)	Conversion (%)			Selectivity (%)								
	Amb-15	S-200	S-100	Amb-15			S-200			S-100		
				MPGEs	DPGEs	TPGE	MPGEs	DPGEs	TPGE	MPGEs	DPGEs	TPGE
8	59.54	0	0	43.89	6.40	9.25	0	0	0	0	0	0
16	77.06	17.53	5.77	46.95	4.83	25.28	15.19	2.34	0	5.77	0	0
24	100	21.74	17.36	24.99	20.84	54.17	14.43	6.25	1.06	12.71	4.65	0
40	100	56.27	42.14	19.82	5.57	74.60	38.25	0.91	17.11	30.74	0.43	10.97
48	100	58.43	57.11	0	0	100	42.80	0.88	14.75	42.35	0.73	14.03
72	—	72.43	69.92	—	—	—	51.61	6.07	14.75	45.27	2.88	21.77

Data are shown as the mean ± 3.08 SD derived from 3 independent samples.

**Table 2:** Product distribution for the etherification of glycerol with 1-butene on Amberlyst-15 and S-200 catalysts [glycerol : 1-butene = 1 : 4, catalyst 10 wt% based on glycerol, at 100°C].

Time (h)	Conversion (%)		Selectivity (%)									
	Amb-15	S-200	Amb-15					S-200				
			1-MBGE	2-MBGE	1,3-DBGE	1,2-DBGE	Dimer	1-MBGE	2-MBGE	1,3-DBGE	1,2-DBGE	Dimer
8	24.76	0	19.78	3.23	0	0	1.75	0	0	0	0	0
16	46.87	9.62	34.85	3.86	3.60	2.64	1.92	6.86	1.94	0	0	0.82
24	58.83	15.57	46.01	7.28	1.46	1.70	2.38	11.77	2.19	0	0	1.61
40	78.71	21.58	53.73	8.09	5.53	6.57	4.79	16.15	2.88	0.38	0.28	1.89
48	80.45	22.72	49.43	5.52	10.55	10.06	4.89	16.42	2.99	0.38	0.29	2.64
72	100	38.99	66.29	7.80	9.71	10.89	5.31	28.19	4.35	1.48	1.27	3.70

Data are shown as the mean ± 1.05 SD derived from 3 independent samples.

**Table 3:** Cetane number of diesel and palm biodiesel after adding 10% of the oxygenated additives.

Fuel	Density (g/cm^3^) at 15°C	CN
Diesel	0.8145	55.0
Diesel + ethanol	0.8133	54.9
Diesel + MTBE	0.8121	56.4
Diesel + PGEs	0.8203	52.0
Diesel + TPGE	0.8188	49.3
Diesel + BGEs	0.8260	52.8
B100	0.8241	67.5
B100 + ethanol	0.8199	69.0
B100 + MTBE	0.8166	70.2
B100 + PGEs	0.8270	66.2
B100 + TPGE	0.8245	67.1
B100 + BGEs	0.8293	66.7

Data are shown as the mean ± 1.08 SD derived from 3 independent samples.

**Table 4:** Octane number of gasoline after adding 10% of the oxygenated additives.

Fuel	Density (g/cm^3^)	RON	MON
Gasoline	0.7553	81.1	91.0
Gasoline + ethanol	0.7504	84.4	94.4
Gasoline + MTBE	0.7497	85.4	96.5
Gasoline + PGEs	0.7585	81.6	94.0
Gasoline + TPGE	0.7578	82.1	93.6
Gasoline + BGEs	0.7603	84.4	98.1

Data are shown as the mean ± 0.23 SD derived from 3 independent samples.
